# Approaches To Managing Relapsed Myeloma: Switching Drug Class or Retreatment With Same Drug Class?

**DOI:** 10.1007/s12288-025-02008-9

**Published:** 2025-03-29

**Authors:** R. Vijjhalwar, A. Kannan, C. Fuentes-Lacouture, K. Ramasamy

**Affiliations:** 1 University Hospital Southampton NHS Foundation Trust, Southampton, UK; 2 Medical Sciences Division, University of Oxford, Oxford, UK; 3 Hospital Militar Central, Bogotá, D.C Colombia; 4 Oxford Translational Myeloma Centre, NDORMS, University of Oxford, Oxford, UK; 5 Oxford University Hospitals NHS Foundation Trust, Oxford, UK

**Keywords:** Retreatment, Relapsed Myeloma, BCMA Targeted Therapies, Biochemical Relapse

## Abstract

Multiple myeloma (MM) is the second most common haematological malignancy characterised by clonal proliferation of plasma cells within the bone marrow. Despite advances in treatment options, myeloma remains incurable. Relapsed MM poses significant challenges due to clonal evolution, drug resistance, patient comorbidities and therefore complexities of therapeutic decision-making. A critical question in managing relapsed MM is whether to switch drug classes or retreat with agents within the same class. The question is present with the recent addition of three new drug classes (XPO1 inhibitors, BCMA targeted agents, GPRC5d targeted agents) to clinical practice. Switching drug classes offers the potential to target alternative disease pathways and introduce new mechanisms of action, which can be particularly beneficial in cases of limited response to previous therapies. On the other hand, retreatment within the same drug class remains an effective strategy for some patients, particularly those who previously achieved durable responses and known tolerability profile. Both approaches require careful consideration on a background of patient-specific factors such as age, comorbidities, disease burden, and quality of life. We discuss the clinical vignette of a 71-year-old male with biochemical relapse after lenalidomide maintenance; this review explores the nuanced decision-making process involved in selecting the most appropriate treatment. By taking a personalised approach and integrating the evolving therapeutic landscape with real-world considerations, this review highlights strategies to optimise outcomes while maintaining tolerability and quality of life in patients with relapsed MM.

## Introduction

Multiple Myeloma (MM) is a malignancy of plasma cells, accounting for approximately 10% of haematological cancers globally making it the second most common haematological cancer [1, 2]. The disease is characterised by the clonal proliferation of plasma cells within the bone marrow, resulting in the overproduction of monoclonal immunoglobulins and systemic complications such as anaemia [3], renal dysfunction [4], bone lesions [5], and hypercalcaemia [5]. While the incidence of MM continues to rise, particularly in ageing populations [6], advancements in therapeutic options have led to substantial improvements in survival outcomes. Between 2012 and 2018, 5-year survival rates in the USA and Europe approached 60% [7, 8], a significant increase compared to the 25% observed in the mid-1970s [9]. Nevertheless, MM remains incurable, with most patients experiencing serial relapses until their disease becomes refractory to available antimyeloma therapies.

Relapsed MM presents distinct clinical challenges. With each successive relapse, the disease accumulates genomic and epigenetic alterations that contribute to clonal heterogeneity, increased drug resistance, and progressively shorter remissions. Modern management of relapsed MM is further complicated by the availability of diverse therapeutic options, including proteasome inhibitors (PIs), immunomodulatory drugs (IMiDs), monoclonal antibodies, and novel therapies such as XPO1 inhibitor, bispecific antibodies and CAR-T cell therapy targeting BCMA and GPRC5d. This has introduced new complexities in clinical decision-making, particularly when determining whether to switch drug classes or retreat with the same class [10]. 

This review aims to address the decision to switch drug classes or retreat with the same class in relapsed MM. To ground this discussion in clinical practice, we include a representative clinical vignette that highlights the complexities of relapse management. By using this case as a foundation, we evaluate data from clinical trials, real-world studies, and disease biology to provide practical insights for optimising patient outcomes.

### Clinical Vignette of Relapsed Multiple Myeloma

A 71-year-old male with International Staging System (ISS) Stage II IgG kappa multiple myeloma and R-ISS 2 with a gain 1q21 on MM FISH was initially treated with a combination of lenalidomide, bortezomib, and dexamethasone, achieving a very good partial response. Following induction, he underwent autologous stem cell transplantation and remained on lenalidomide maintenance therapy for a fixed duration of two years. During a routine follow-up (approximately 4 years from diagnosis), serum protein electrophoresis revealed an increase in M-spike from 2 g/L to 7 g/L, alongside a rise in the serum free light chain ratio to 40 (excess kappa light chains). This constitutes a biochemical relapse as per IMWG response criteria [11]. Blood tests, including serum calcium, renal function (serum creatinine and estimated glomerular filtration rate), and haemoglobin, were within normal ranges. Imaging with whole-body low-dose CT showed no evidence of new or worsening lytic bone lesions. The patient remained asymptomatic, with no findings to suggest clinical disease progression.

### Definitions of Relapsed MM

Relapse in MM often manifests as either biochemical relapse before a variable transition in time to clinical relapse. Biochemical relapse is defined as disease progression detected solely through laboratory markers, without any evidence of clinical symptoms or organ damage. The International Myeloma Working Group (IMWG) provides specific criteria to diagnose biochemical relapse, including a doubling of the M-component (monoclonal protein) in serum or urine across two consecutive tests separated by at least two months, with a baseline M-protein level of ≥ 5 g/L [11]. Alternatively, biochemical relapse may also be diagnosed if there is a significant increase in other disease markers, such as an absolute rise in serum M-protein of ≥ 10 g/L, an increase in urine M-protein of ≥ 500 mg/24 hours, or an increase in serum-free light chains (FLC) by ≥ 20 mg/dL or 25%, whichever is greater [11]. 

Beyond relapse type, refractory disease is also classified by the number of drug classes to which the disease has become resistant. Refractory MM is defined as disease that fails to respond, either by progressing or not achieving at least a minimal response to the most recent line of therapy, or that progresses within 60 days of stopping treatment. Single-refractory MM refers to resistance to one major drug class, such as a PI or an IMiD. Double-refractory MM describes resistance to both a PI and an IMiD. Triple-refractory MM occurs when the disease is resistant to at least one PI, one IMiD, and an anti-CD38 monoclonal antibody. Finally, penta-refractory MM represents a challenging scenario, where the disease is resistant to two PIs, two IMiDs, and an anti-CD38 antibody, leaving very few remaining treatment options.

On the other hand, clinical relapse reflects symptomatic progression of the disease with features of end-organ damage causing hypercalcemia, renal dysfunction, anaemia, and bone disease. Clinical relapse can also be marked by recurrent infections and worsening comorbidities.

In our clinical vignette, the 71-year-old male presents with biochemical relapse, indicated by a > 5 g/L increase in M-protein without any evidence of end-organ damage. This raises an important clinical question: should treatment begin now, or is it better to wait until clinical relapse develops?

### When Should this Patient Begin Treatment for Relapsed MM?

There is currently no uniform approach on whether MM patients experiencing a biochemical relapse should be treated immediately or whether therapy should be delayed until clinical relapse. This is largely driven by clinician and patient preferences, available drug therapies and the heterogeneity among patients in biochemical relapse treated in clinical practice and in clinical trials. Many of the pivotal trials that have led the approval of drugs within relapsing MM have inclusion criteria that requires a patient to be in either biochemical or clinical relapse in order to be included within the trial. However, the European Myeloma Network (EMN) advocates for treating biochemical relapse as a non-aggressive form of clinical relapse, provided it aligns with the IMWG criteria [12, 13]. Commencing treatment during biochemical relapse in MM rather than deferring treatment until clinical relapse manifests has shown to produce superior outcomes in non-randomised studies [14, 15]. Patients receiving treatment at the stage of biochemical relapse prior to clinical relapse exhibit prolonged median overall survival (OS) and progression-free survival (PFS) compared to those treated at clinical relapse or those with a concurrent biochemical and clinical relapse, including cases with extramedullary disease [15]. Additionally, patients with biochemical relapse had longer median time from second-line treatment to the next treatment compared with patients who had a clinical relapse [14]. Efficacy of treatment with agents including Bortezomib and Dexamethasone, Carfilzomib and Dexamethasone or Lenalidomide and Dexamethasone (measured by response rates) is comparable whether it is initiated at biochemical relapse or clinical relapse showing that timely intervention at biochemical relapse does not compromise treatment efficacy [16–18]. Moreover, the tolerability of salvage treatments is likely to be better when administered at biochemical relapse compared with a clinical relapse, when tumour and symptom burden is higher and there is resultant end-organ damage. There may also be related to the greater flexibility in treatment dosage required when intervening early at biochemical relapse, resulting in better tolerability, compared with initiating treatment in clinical relapse when organ damage has occurred. But advocates of treatment at clinical relapse will argue the lack of randomised trial evidence in this space. They will also point to a proportion of patients having an indolent relapse pattern and may be able to avoid early reintroduction of antimyeloma therapy. This is a reasonable challenge, therefore patients relapsing should undergo a thorough evaluation for high-risk relapse factors where the initial biochemical relapse heralds an aggressive relapse. Blood film examination for circulating plasma cells, extra medullary disease on whole body imaging, heavy marrow infiltration and presence of high-risk genomic features are indicative of a rapid conversion of biochemical to clinical relapse. In addition, relapsing on antimyeloma agents and shorter time from diagnosis to first relapse are predictors of an aggressive disease course [19]. 

Hence, relating back to the clinical vignette presented, the absence of symptoms or organ damage provides an opportunity for timely treatment, which has been shown to improve PFS and OS compared to waiting for clinical relapse. Initiating therapy at this stage could optimise outcomes while maintaining tolerability, as the tumour burden remains low and complications are minimal. It aligns with the European Myeloma Network’s view that biochemical relapse can be considered a non-aggressive form of clinical relapse warranting early intervention. If patient requests closer monitoring, a thorough evaluation with blood tests, whole body imaging and marrow evaluation including genomics should be performed to evaluate the risk of early evolution to clinical relapse.

### What Should be the Treatment Goals in this Patient?

The first relapse of MM typically occurs around 3–4 years after diagnosis, with subsequent remissions becoming progressively shorter as the disease becomes more aggressive, driven by an increasing proportion of treatment-resistant myeloma clones [20–22]. The primary goal of MM treatment is to achieve a maximal response, which is closely linked to better disease control and prolonged survival [23]. Across all stages of treatment, deep responses are often sustained and is associated with longer survival [23]. Key treatment objectives include effective disease control, often indicated by achieving a complete response (CR), very good partial response (VGPR) or better and minimal residual disease (MRD) negativity. More recently, MRD negativity has been reported in a proportion of relapsed MM patients on effective therapies [24]. But due to the complexity of MM biology, response to therapy can also be variable. Hence, a small proportion of patients achieve good initial responses that may be short-lived, whereas others achieve only minimal responses (MRs) that are durable and clinically relevant [25, 26]. 

Achieving a deep response significantly reduces the burden of malignant cells, especially at the start of treatment; however, this must be balanced with patient tolerability, quality of life, and preferences [27]. Treatment strategies must carefully consider toxicities, age, and patient-specific factors such as frailty to optimise outcomes. Many anti-myeloma agents are associated with significant side effects, including hematologic toxicities, peripheral neuropathy, gastrointestinal issues, and cardiotoxicity, which can lead to premature treatment discontinuation. For instance, IMiDs such as lenalidomide can lead to neutropenia [28, 29] with attendant infection risk and an possible increased risk of thromboembolism [30] and risks are further heightened in the presence of renal impairment [31]. Similarly, proteasome inhibitors particularly carfilzomib are associated with a heightened risk of cardiotoxicity [32]. Therefore, strategies such as attenuated schedules, liberal dose adjustments, use of supportive medications, and fixed duration therapeutic approaches can help mitigate these toxicities.

For older or frail patients, less intensive regimens or adjusted dosing schedules may be necessary to avoid cumulative toxicity while maintaining quality of life. The IMWG frailty score predicts mortality and toxicity in elderly MM patients by assessing age, comorbidities, and cognitive and physical health [33]. The score categorises patients into three groups—fit, intermediate, and frail—with frail patients having lower survival rates and more adverse events. Hence, comorbidities, frailty, and performance status must be factored into treatment decisions, as elderly or frail patients may benefit more from tailored, less aggressive approaches.

In addition, patients who are classified as high-risk, based on an genomic and clinical criteria, may be treated differently to those with indolent MM [2, 12, 34]. High-risk patients include those with adverse cytogenetics such as [del17p, t(4;14), add 1q/del1p, t(14;16), high-risk gene expression profile, high β-2-M], low-albumin or high-serum LDH [35]. High-risk patient groups often require triplet drug regimens to achieve adequate disease control. Patients with standard-risk or indolent disease can be treated with less-intensive regimens.

Taken together, treatment decisions should be highly individualised, considering prior treatments, cytogenetic profiles, patient preferences, and overall treatment goals. While a deep response is desirable, a balanced approach is essential, particularly in cases where the risks of intensive therapy may outweigh its benefits. The focus should remain on achieving long-term disease control while maintaining quality of life.

### What Therapeutic Options are Available for this Patient in Relapsed MM?

Our patient has responded to therapy and has been exposed to proteasome inhibitor for a short period and had exposure to 2 years of lenalidomide therapy during maintenance. The clinical history and bloods suggest an indolent relapse.

#### Immunomodulatory Drugs (IMiDs)

IMiDs, including thalidomide, lenalidomide, and pomalidomide, have demonstrated substantial benefits in improving response rates, PFS, and OS in relapsed MM [36, 37]. IMiDs function by binding to cereblon (CRBN), leading to the degradation of transcription factors Ikaros (IKZF1) and Aiolos (IKZF3) [38, 39]. This mechanism disrupts myeloma cell proliferation and enhances anti-tumour immunity through activation of natural killer (NK) and T cells [40]. 

IMiDs are typically used in combination regimens. Lenalidomide combined with dexamethasone achieved an overall response rate (ORR) of 76%, which increases to 93% when daratumumab is added, as demonstrated in studies of the daratumumab, lenalidomide, and dexamethasone (DRd) regimen [41, 42]. Similarly, pomalidomide is effective in patients who are refractory to lenalidomide and bortezomib, often combined with dexamethasone [43–45] and monoclonal antibodies (mAbs) such as daratumumab [46] or isatuximab [47]. 

In first relapse, a combination of proteasome inhibitor and IMiD-based regimens such as carfilzomib, lenalidomide, and dexamethasone (KRd) [48] or ixazomib, lenalidomide, and dexamethasone (IRd) [49] have been shown to be effective. KRd achieves an ORR of approximately 87%, with a median PFS of 26.3 months compared to 17.6 months with lenalidomide and dexamethasone alone [48]. In later relapses, pomalidomide-based regimens maintain efficacy, providing ORRs of 30–60% in refractory patients and a median OS of approximately 12–15 months, depending on patient characteristics [46, 47, 50]. In addition, IMiDs have also shown efficacy in partially overcoming high-risk cytogenetic abnormalities. In combination with proteasome inhibitors or mAbs, IMiDs show better efficacy in high risk del(17p) and t(4;14) patients. In the POLLUX trial, DRd achieved a 60% reduction in the risk of progression or death compared to lenalidomide and dexamethasone alone in high-risk patients [42]. 

#### Proteasome Inhibitors (PIs)

PIs are small-molecule drugs target the proteasome, a cellular complex responsible for degrading ubiquitinated proteins. In MM cells, which have a high rate of immunoglobulin synthesis, the proteasome and the unfolded protein response (UPR) play critical roles in managing protein folding and turnover [51–53]. The 26 S proteasome, composed of a 19 S regulatory particle and a 20 S catalytic core, contains three catalytic subunits—β1, β2, and β5—responsible for caspase-, trypsin-, and chymotrypsin-like activities, respectively [51–53]. Inhibiting these catalytic sites leads to protein accumulation, endoplasmic reticulum (ER) stress, and apoptosis. PIs block this essential protein degradation pathway, leading to intracellular protein accumulation and apoptosis.

Bortezomib, the first generation PI, has shown significant efficacy in relapsed MM, even in patients with renal impairment, with improved outcomes in registrational trial APEX (ORR: 38% vs. 18%; TTP: 6.2 vs. 3.5 months; OS: 80% vs. 66%) [36]. Subcutaneous bortezomib improves tolerability, and it is frequently used in combination therapies [54]. Carfilzomib, a next-generation PI, demonstrated superior progression-free survival (PFS) over bortezomib in the ENDEAVOR trial [55] (18.7 vs. 9.4 months) and further improved outcomes in the ASPIRE trial [48] when combined with lenalidomide and dexamethasone (ORR: 87.1%; PFS: 26.3 months). Ixazomib, an oral PI, also showed improved PFS (20.6 vs. 14.7 months) when added to lenalidomide and dexamethasone in the TOURMALINE-MM1 trial [49]. 

#### Monoclonal Antibodies (mAbs)

Drug resistance and a permissive bone marrow microenvironment are key events in MM pathogenesis. Whilst IMiDs and PIs are able to target this to some extent, monoclonal antibodies (mAbs) such as Elotuzumab, Daratumumab, and Isatuximab have emerged as a strategy to directly overcome the immunosuppressive MM tumour microenvironment and promote anti-tumour immunity [56]. 

Elotuzumab targets SLAMF-7, a surface protein expressed on more than 95% of bone marrow myeloma cells [57, 58]. Although it has limited single-agent activity in relapsed MM, combining elotuzumab with lenalidomide and dexamethasone (Rd) yields an ORR of over 80% [59]. The ELOQUENT-2 trial showed superior progression-free survival (PFS) at 3 years (27% vs. 19%), with an ORR of 78.5% compared to 65.5% for Rd alone [59]. PFS benefits were observed regardless of age, prior therapies, or cytogenetic risk, and elotuzumab has also shown activity with pomalidomide and dexamethasone in the ELOQUENT-3 trial [60]. 

Daratumumab, an anti-CD38 mAb, induces cell death via antibody-dependent cytotoxicity and complement activation [61]. It has potent monotherapy activity, with a 42% ORR in refractory patients, and performs synergistically in combinations. The POLLUX trial demonstrated improved ORR (93% vs. 76%), complete response (CR) rate (46% vs. 20%), and 18-month PFS (79% vs. 49%) with daratumumab-Rd compared to Rd alone [41]. The CASTOR trial showed similar advantages with daratumumab-bortezomib-dexamethasone (Vd) over Vd alone (12-month PFS: 60% vs. 22%; ORR: 84% vs. 63%) [42]. Daratumumab has been approved for use with Rd, Vd, pomalidomide-dexamethasone (Pd), and carfilzomib-dexamethasone (Kd), and more recently, subcutaneous formulations of daratumumab has been approved which enhance convenience [62]. 

Isatuximab, another anti-CD38 mAb, is approved for use with pomalidomide and dexamethasone (POM-DEX) in patients with ≥ 2 prior therapies, and with carfilzomib-dexamethasone (CFZ-DEX) in those with 1–3 prior treatments. The ICARIA-MM trial [47] showed improved PFS with isatuximab-POM-DEX (11.5 vs. 6.5 months), while the IKEMA study [63] showed superior efficacy in combination with CFZ-DEX (ORR: 87% vs. 83%).

#### Antibody Drug Conjugates

Another modality used in relapsed MM are antibody-drug conjugates (ADCs) designed to attack B-cell maturation antigen (BCMA) [64]. Belantamab mafodotin, a BCMA-targeting IgG humanized ADC, has demonstrated efficacy in phase 3 trials when combined with either a PI or an IMiD as a second-line therapy after first relapse [65, 66]. 

The DREAMM-7 trial compared belantamab mafodotin, bortezomib, and dexamethasone (BVd) with daratumumab, bortezomib, and dexamethasone (DVd) in patients with relapsed MM who had received at least one prior line of therapy [65]. After a median follow-up of 28.2 months, the median PFS was significantly longer in the BVd group (36.6 months, 95% CI: 28.4–NR) compared to the DVd group (13.4 months, 95% CI: 11.1–17.5), with a HR for disease progression or death of 0.41 (95% CI: 0.31–0.53, *P* < 0.001). Additionally, more patients in the BVd arm achieved deeper responses [65]. 

Similarly, the DREAMM-8 trial evaluated belantamab mafodotin, pomalidomide, and dexamethasone (BPd) versus pomalidomide, bortezomib, and dexamethasone (PVd) in lenalidomide-exposed patients after at least one prior line of therapy. The 12-month estimated PFS was 71% in the BPd arm compared to 51% in the PVd arm (HR: 0.52, 95% CI: 0.37–0.73, *P* < 0.001). However, overall survival data is not yet available [66]. 

Notably, the US FDA withdrew its approval of Belantamab mafodotin over concerns about its efficacy compared to standard-of-care regimens. However, the ongoing trials indicate its potential in combination therapies, and reapproval is expected pending further positive outcomes, with the decision expected by 23rd July 2025.

A more recent ADC in development is AZD0305, a first-in-class GPRC5D-targeting ADC. This investigational therapy is currently being evaluated in a modular phase I/II trial for relapsed/refractory MM patients who have received at least three prior lines of therapy, including a PI, an IMiD, and an anti-CD38 antibody. Patient recruitment is ongoing, and trial results are awaited [67]. 

#### Chimeric Antigen Receptor (CAR) T-cell Therapy

CAR T-cell therapy has emerged as a promising treatment for relapsed MM, particularly in patients with triple-class exposed disease. Two target B-cell maturation antigen (BCMA)-targeted CAR T-cell therapies, ciltacabtagene autoleucel (cilta-cel) and idecabtagene vicleucel (ide-cel), have received conditional approval based on phase 1b/2 clinical trials. CAR T-cell therapies primarily target BCMA, although other targets such as GPRC5D [68] and CD19 [69] are also being investigated.

Cilta-cel has shown an ORR of 98% and a stringent complete response (sCR) rate of 82% in heavily pre-treated patients, with 12-month progression-free survival (PFS) rates of 76% in the CARTITUDE-4 trial [70]. Ide-cel has demonstrated an ORR of 81% and a complete response (CR) rate of 39%, with the KarMMa-3 trial reporting a 71% ORR and 39% CR in patients with 2–4 prior lines of therapy [70]. Emerging CAR T-cell therapies, such as ARI0002h [71], ddBCMA [72], and GC012F [73], have shown promising response rates in clinical trials, with GC012F achieving a 93% ORR in relapsed MM.

One notable concern with BsAb therapy is the increased risk of hypogammaglobulinemia due to profound B-cell depletion, predisposing patients to recurrent infections [74, 75]. Intravenous immunoglobulin (IVIG) prophylaxis is recommended in patients with severe hypogammaglobulinemia (< 4 g/L) or those experiencing recurrent infections. Given the sustained immunosuppressive effects of BsAbs, IVIG supplementation may be required throughout treatment to mitigate infection risk and improve long-term tolerability.

#### Bispecific Antibodies

Bispecific antibodies (BsAbs) represent a novel immunotherapy that work by binding simultaneously to a target on myeloma cells and to CD3 on T-cells. This causes activation of T-cells which lead to the direct killing of the myeloma cells [73]. Validated targets on the surface of myeloma cells include BCMA, GPRC5D [76], and FcRH5 [77]. Similar to CAR T-cell therapy, these are currently licensed in relapsed and refractory MM, with combinations trials and upfront investigation ongoing.

Teclistamab, a BCMA-CD3 bispecific antibody, has been approved for relapsed MM and has demonstrated an ORR of 63% and a CR rate of 39.2% [78]. Other BsAbs, such as Elranatamab and linvoseltamab, have shown encouraging PFS rates of 51% at 15 months and 69% at 12 months, respectively [79, 80]. Talquetamab, targeting GPRC5D and CD3, achieved an ORR of 74% with a CR rate of 34% in RRMM [76]. 

Unlike CAR T-cell therapy, which involves a single infusion, BsAbs are typically administered continuously via subcutaneous or intravenous routes, with the potential for at-home administration, reducing treatment burden. Indirect comparisons suggest that BCMA-targeted therapies, including cilta-cel, ide-cel, and teclistamab, offer robust outcomes in triple-class exposed RRMM patients. Other BsAbs in development include cevostamab [81], which targets FcRH5 and CD3, and linvoseltamab, which targets BCMA and CD3, showing deep and durable responses [80]. 

#### Exportin-1 (XPO1) Inhibitors

XPO1 inhibitors have emerged as a novel treatment strategy for relapsed or refractory multiple myeloma, particularly in patients with triple-class refractory disease. XPO1, a nucleocytoplasmic shuttling protein, is overexpressed in multiple myeloma, playing a critical role in exporting tumour suppressor proteins (TSPs) and oncoprotein mRNA from the nucleus to the cytoplasm. Selinexor, the first-in-class XPO1 inhibitor, blocks this export, leading to the nuclear retention of tumour suppressors such as p53, FOXO, and IκB, thereby restoring their anti-proliferative and pro-apoptotic functions. Selinexor is orally bioavailable, making it a convenient option for patients with heavily pre-treated RRMM. The phase 3 BOSTON trial demonstrated improved PFS (13.9 months vs. 9.5 months) with the combination of Selinexor, bortezomib, and dexamethasone compared to standard bortezomib and dexamethasone, while reducing treatment-related neuropathy by lowering bortezomib exposure [82]. Selinexor has also been evaluated in combination with lenalidomide and dexamethasone, showing promising efficacy in patients with RRMM, particularly those without prior lenalidomide resistance [83]. 

### Should we Treat this Patient Using Combination Therapy or Monotherapy?

In the management of relapsed MM, combination therapy has consistently emerged as the preferred approach over single-agent regimens due to its superior efficacy in improving patient outcomes across many randomised controlled trials in RRMM. Combination therapy addresses relapsed myeloma biology challenges by targeting multiple pathways in myeloma cells, helping to overcome resistance and extend PFS. Several trials have demonstrated that triplet therapies significantly improve PFS compared to single-agent or doublet regimens [47, 48, 59, 63, 65, 84]. For instance, lenalidomide combined with dexamethasone (Rd) has been shown to improve ORR, duration of response, and TTP compared to dexamethasone alone in the MM-009 and MM-010 trials [41]. Novel combinations, including carfilzomib, ixazomib, elotuzumab, or daratumumab with Rd or bortezomib and dexamethasone (Vd), have shown substantial activity, even in patients with high-risk cytogenetics. Meta-analyses further highlight the effectiveness of daratumumab-based regimens, with daratumumab plus Rd identified as having the highest probability of improving PFS across relapsed myeloma patient subgroups [85]. 

Specific regimens such as VRd (bortezomib, lenalidomide, and dexamethasone) and IRd (ixazomib, lenalidomide, and dexamethasone) have proven effective and well-tolerated, particularly in patients with high-risk cytogenetic abnormalities [49, 86]. Isatuximab combinations, such as with pomalidomide and dexamethasone (IPd) [47, 63] or carfilzomib and dexamethasone (IKd) [63], have shown superior PFS compared to doublet therapies in lenalidomide refractory patients, while daratumumab-based regimens, including DRd and DVd, are integral components of modern triplet and quadruplet treatment strategies in early relapse myeloma patients.

Despite the clear advantages of combination therapy, single-agent or doublet regimens may still have a role in specific clinical scenarios. In frail or elderly patients, where minimising toxicity is paramount, regimens such as pomalidomide plus dexamethasone may be appropriate.

Hence, relating back to the clinical vignette presented, the patient’s relapsed MM highlights the need for an effective approach that prioritises combination therapy over single-agent regimens. Clinical evidence strongly supports the use of triplet regimens in relapsed MM due to their superior efficacy in improving PFS and ORR compared to single agents or doublets. In this case, a combination therapy targeting multiple pathways in myeloma cells would be the preferred strategy to overcome resistance and maximise treatment outcomes.

### What Investigations Will the Patient Need before Initiation of Treatment?

Refer to Table 1.

**Table 1:** Showing the panel of tests recommended pre-initiation of relapse treatment for MM

Category	Test	Purpose	Key Considerations and Associated Agents
**Performance Status**	ECOG	To determine functional status and guide treatment intensity.	Frail patients may be better suited for doublet regimens like Rd or Pd.
**Blood Tests**	FBC	To assess for anaemia, thrombocytopenia, or other cytopenias.	Thrombocytopenia is common with lenalidomide, pomalidomide, and bortezomib.
	Serum protein electrophoresis	To quantify M-protein for disease burden monitoring.	Essential for establishing a baseline to be able to check response to treatment in relapsed MM.
	Serum free light chain assay	To evaluate free light chain levels, especially in non-secretory MM or light chain disease.	Crucial for establishing a baseline.
	Renal profile (urea, creatinine, eGFR)	To assess kidney function, as IMiDs are primarily renally excreted.	Dose adjustments is needed for iMIDs such as lenalidomide and pomalidomide which are primarily renally excreted.
	Beta-2 microglobulin	Prognostic marker and combined with serum albumin, it is used for ISS staging.	High levels predict worse outcomes and influence treatment intensity decisions.
	Serum albumin	Used with beta-2 microglobulin for ISS staging and prognosis.	Relevant for initial risk stratification in all therapeutic approaches.
	Prothrombin time (PT) and aPTT	To evaluate clotting function and VTE risk.	Relevant for IMiDs (e.g., lenalidomide, pomalidomide), which may require anticoagulation.
	Blood group typing	To evaluate and document blood group.	Daratumumab can interfere with blood compatibility testing. Blood testing should therefore be done before initiating therapy to avoid transfusion complications.
**Urine tests**	24-hour urine protein quantification	To establish baseline renal function, detect Bence Jones proteinuria, and evaluate marker of disease burden.	Enables monitoring of relapse and in detecting early kidney dysfunction.
**Bone Marrow Examination**	Aspirate and biopsy	To confirm marrow involvement and plasma cell infiltration.	Guides escalation to triplet or high-intensity therapies like carfilzomib-based regimens.
	Cytogenetics and FISH	To identify high-risk abnormalities (e.g., Chromosome 1 changes, del(17p), t(4;14), t(14;16)).	High-risk cytogenetics influence choice of agents like daratumumab or KRd.
**Imaging**	PET-CT	For detecting extramedullary disease and response assessment.	Crucial for CAR T-cell or bispecific antibody therapies.
**Infection Screening**	Hepatitis B and C, HIV	To evaluate infection risks before immunosuppressive therapy, including mAbs.	Reactivation prophylaxis needed for daratumumab or isatuximab.
	CMV and EBV serology	To assess risk of viral reactivation	Relevant for CAR T-cell and bispecific antibody therapies.
**Cardiac Assessment**	Echocardiography	To assess baseline cardiac function	Carfilzomib is associated with cardiotoxicity.
**Neurological Assessment**	Baseline neurological exam	To evaluate peripheral neuropathy risk.	Essential for agents like bortezomib, thalidomide, and ixazomib, which are associated with peripheral neuropathy.
**Pulmonary Function**	Pulmonary function testing	To assess lung function	Cyclophosphamide and some conditioning regimens may impact pulmonary reserve.

### How should we Treat this Patient: Switch Drug Classes or Re-Treat with another Drug within the Same Drug Class?

#### Rationale for Switching Drug Classes

**Fig. 1:**
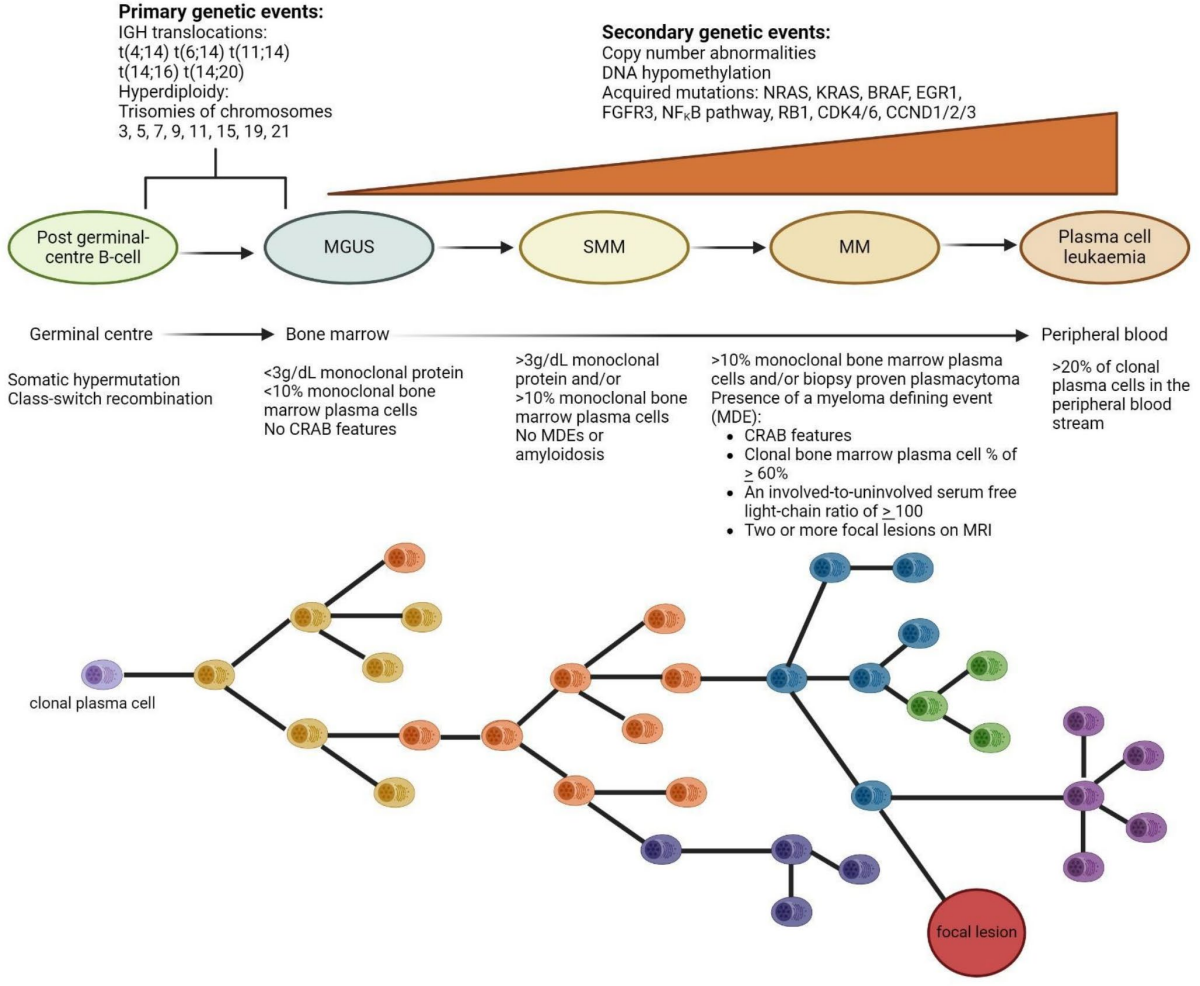
Multiple Myeloma pathogenesis- branched clonal evolution and genetic events. Adapted from Van de Donk et al., [1]

Over the course of MM treatment, disease progression is driven by the emergence of sub-clonal populations with unique genetic profiles and varying sensitivities to therapy. These sub-clones evolve under the selective pressure of treatment, leading to drug resistance and shifting clonal dominance (refer to Fig. 1) [20]. As a result, successive lines of therapy are often characterised by diminishing duration of response, PFS, and OS. This evolving clonal landscape underscores the importance of switching drug classes to target distinct pathways and overcome resistance mechanisms.

The tumour microenvironment also contributes to drug resistance in relapsed MM. Myeloma cells exploit stromal and immune cells to create a supportive niche, while immune dysfunction—mediated by mechanisms such as myeloid-derived suppressor cells and altered T-cell populations—further suppresses anti-tumour immunity [56, 87]. These interactions reduce the efficacy of therapies over time, necessitating the introduction of agents with novel mechanisms of action to restore disease control.

Drug resistance to established classes such as IMiDs and proteasome inhibitors is a key driver for switching therapies. IMiD resistance is often associated with mutations in cereblon (CRBN) or Ras pathway genes, while PI resistance may result from impaired proteasome function or altered protein degradation pathways [51, 52, 88]. Similarly, resistance to anti-CD38 monoclonal antibodies like daratumumab is frequently linked to decreased CD38 expression due to endocytosis, trogocytosis, or microvesicle release [61]. Switching drug classes allows clinicians to bypass these resistance mechanisms and re-establish disease control.

High-risk cytogenetic abnormalities, such as del(17p), t(4;14), and gain 1q, further highlight the need for switching therapies in relapsed MM. Patients with these abnormalities often require combination regimens involving monoclonal antibodies, BCMA-targeted therapies, or other novel agents to address their unique vulnerabilities. In addition to cytogenetic risk, the Revised International Staging System (R-ISS) refines prognostic stratification by incorporating β2-microglobulin, LDH, and high-risk cytogenetics [89]. Patients classified as R-ISS III typically have more aggressive disease with poorer outcomes, necessitating early introduction of therapies with novel mechanisms of action, such as BCMA-targeting CAR T-cell therapy (e.g., ide-cel, cilta-cel) or bispecific antibodies (e.g., teclistamab, elranatamab) [89]. In contrast, R-ISS I patients may have a more indolent relapse, where retreatment within the same drug class could still be effective. R-ISS II, as the patient in our clinical vignette, represents a heterogeneous group, where treatment selection should be individualised based on prior therapy duration, cytogenetics, and β2-microglobulin levels.

Another factor to consider is β2-microglobulin levels, which serves as a surrogate for tumour burden and disease progression [90, 91]. A rapid rise in β2-microglobulin levels at relapse suggests high disease activity and potential resistance to previously used regimens, strengthening the rationale for switching drug classes. In patients with increasing β2-microglobulin levels who relapse shortly after IMiD-based maintenance therapy, switching to a monoclonal antibody or a proteasome inhibitor is often favoured over continued reliance on IMiDs. However, in patients with stable β2-microglobulin levels and a prolonged remission before relapse, retreatment within the same class, such as using pomalidomide after lenalidomide, may still be effective, particularly when combined with a monoclonal antibody.

Additionally, class-switching is often necessary to address patient-specific factors such as comorbidities, end-organ damage, and tolerability to the drug class when administered previously. For instance, cardiovascular disease may limit the use of carfilzomib, neuropathy will limit Bortezomib use, while renal impairment may necessitate significant adjustments to lenalidomide or pomalidomide dosing, making alternative drug classes a safer and more effective choice.

#### Rationale for re-treating With Another Drug Within the Same Drug Class

Retreatment with drugs from the same class remains a viable option for many patients with relapsed MM, particularly when previous therapies were well-tolerated and yielded durable responses of a number of years. Several clinical trials support this approach, demonstrating that retreatment can provide meaningful clinical benefits, especially when resistance mechanisms are absent or can be overcome through dose adjustments or combination therapy. Lenalidomide, Ixazomib and Carfilzomib trials included patients with prior proteasome and lenalidomide exposure [48, 49]. Real world studies show that prior exposure without refractoriness does not affect treatment efficacy of triplet regimens.

Retreatment with lenalidomide, an IMiD, has shown sustained efficacy in relapsed MM. The LENALOGIC trial demonstrated that lenalidomide retreatment improved PFS and ORR in patients who had initially responded to this agent [92]. Similarly, bortezomib has been shown to retain efficacy upon retreatment in certain patient populations. The APEX trial revealed that patients relapsing after prior bortezomib therapy could achieve meaningful clinical responses when re-treated, particularly when bortezomib was combined with dexamethasone to overcome resistance.

When considering re-treatment, a key factor to consider is the duration of the index drug exposure. A recent study found patients with a longer time gap between the initial line of therapy with index drug and retreatment with index drug (> 46.1 months) had better PFS with retreatment (28.2 vs. 8.9 months; *P* = 0.016) [93]. Similarly, patients with a longer time on initial therapy with the index drug (> 28.4 months) had a superior PFS with retreatment (median PFS, 16.9 vs. 8.1 months; *P* < 0.001) [93]. Hence, time from exposure of index drug as well as time on the index drug should be considered when deciding on treatment in relapsed MM. While retrospective analyses provide limited data to establish definitive recommendations for retreatment based on prior exposure duration, these findings suggest that a longer time from initial exposure may enhance the effectiveness of reusing the same agent. This should be considered when making treatment decisions in relapsed multiple myeloma.

Retreatment is also supported by its known and favourable safety profile in certain drug classes. IMiDs like lenalidomide and pomalidomide are well-tolerated in many patients, particularly when combined with low-dose dexamethasone. For example, the MM-015 study found that lenalidomide plus dexamethasone provided durable responses with manageable toxicity in relapsed MM, even when the drug was reintroduced after prior use [92, 94, 95]. Similarly, the ENDEAVOR trial demonstrated that carfilzomib plus dexamethasone was superior to bortezomib plus dexamethasone in bortezomib-exposed patients with relapsed multiple myeloma [55]. 

While resistance mechanisms often limit retreatment efficacy, these barriers can sometimes be mitigated. For instance, adjustments in dosing schedules or the addition of novel agents can overcome resistance to proteasome inhibitors or IMiDs. The MM-009 and MM-010 trials evaluated the combination of lenalidomide and dexamethasone in patients with relapsed MM who had previously received thalidomide. Despite prior exposure, lenalidomide was highly effective, significantly improving PFS and ORR compared to dexamethasone alone. This suggests that lenalidomide’s unique pharmacodynamic properties and combination with dexamethasone allowed it to overcome resistance associated with thalidomide exposure.

Combination approaches with the same drug class have also shown promise in enhancing retreatment efficacy. PI and IMiD combination, PI and monoclonal antibodies or IMiDs with monoclonal antibodies have shown favourable results in relapsed MM [41, 42, 48, 49]. 

Hence, relating back to the clinical vignette presented, the patient had a prior response to lenalidomide, combined with the prolonged remission achieved during maintenance therapy. This underscores the potential utility of retreatment within the same class, particularly if resistance mechanisms are absent or can be mitigated through strategic modifications.

**Fig. 2:**
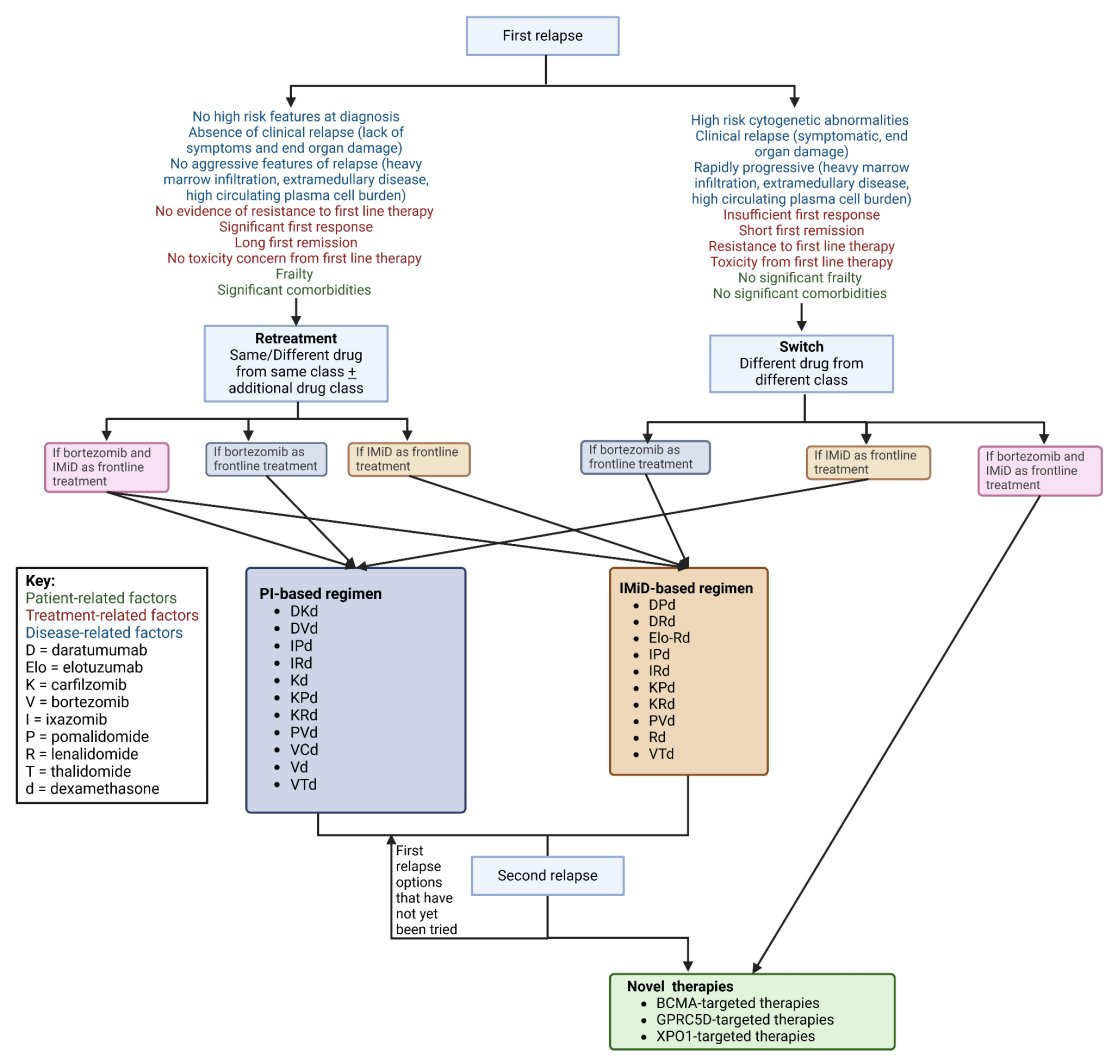
Summarises an algorithm to consider when a patient relapses with MM

However, if relapse had occurred during lenalidomide maintenance therapy, it would raise the possibility of emerging resistance, potentially driven by alterations in CRBN function or downstream pathways. In this context, switching to a new generation drug within the same drug class to incorporate agents with novel mechanisms of action, such as daratumumab or isatuximab, may offer an opportunity to bypass resistance and target distinct aspects of the disease. Figure 2 summarises the algorithm to consider in patients with relapsed MM. Evidence from trials like POLLUX and CASTOR also supports the use of daratumumab-based combinations in lenalidomide-exposed, but not refractory, patients, providing significant improvements in PFS. APOLLO, OPTIMISSM, CANDOR, IKEMA and ICARIA trials provide evidence of use of second-generation PIs and IMiDs in combination with monoclonal antibodies to salvage patients relapsing whilst on earlier generation drugs within the same drug class.

The absence of symptoms or end-organ damage also positions the patient as an ideal candidate for therapies that prioritise tolerability and preserve quality of life. For instance, iMiDs like pomalidomide could be considered, given their efficacy in lenalidomide-refractory cases, as demonstrated in the OPTIMISMM trial.

#### Key Recommendations for this Patient

1. Initiate therapy now rather than delaying until clinical relapse to prevent disease progression and maintain efficacy while tumour burden remains low.
2. If the prior lenalidomide response was long-lasting, DRd, KRD or IRd are reasonable options.
3. If there is concern about lenalidomide resistance, switch to a PI-based regimen like DVd/ DKD or IKd.
4. Monitor for early progression with regular M-protein, FLC, and imaging if opting for close surveillance rather than early treatment if patient prefers that approach.

## Discussion

The biology of multiple myeloma lend to a relapse-remission pattern, and the disease remains incurable despite new treatment strategies. Subsequent lines of treatment generally depend on previously received therapies, and concepts like “lenalidomide refractoriness” — defined as disease progression during active treatment or within the first 60 days after its discontinuation [2] — are among the factors to consider when choosing subsequent treatments during relapse of disease. In this sense, re-exposure to previous medications is a valid option depending on the individual case. However, treatment selection must also consider the patient’s age, functional status, comorbidities, and of course, always aim to provide the best possible quality of life, considering the side effects these treatments may cause for patients as they are received continuously.

In these cycles of relapse-remission, once patients have been exposed to multiple lines of treatment including a PI, an IMiD, and an anti-CD38 antibody, they acquire a state of triple exposure/refractoriness. This, as seen in real-world studies such as MAMMOTH [96] and LOCOMMOTION [97], dramatically impacts the median PFS and OS, associated with a significant deterioration in quality of life. Therefore, clinical decision making in early relapse management requires careful consideration.

In the pursuit of improving outcomes, especially for these triple exposed/refractory patients, new drugs have been developed with mechanisms of action different from existing ones. Unfortunately, the widespread use of these novel therapies is limited by the lack of resources in many countries, including high cost. Even if financial access is secured, the administration of these medications requires the expertise and multidisciplinary approach to manage the most common side effects. In this context, when considering switching drug classes to newer redirected T-cell therapies, it is important to consider the burden of toxicity that these therapies carry.

Both CAR T-cells and BsAbs can lead to haematological and gastrointestinal adverse effects (including neutropenia, anaemia, nausea and diarrhoea), cytokine release syndrome (CRS, a syndrome of systemic inflammation including fever, hypotension, and multi-organ failure) and immune effector cell-associated neurotoxicity (ICANS), secondary to dysregulated immune hyperactivation and cytokine production (Meir et al.). For example, in the KarMMA-3 trial of ide-cel, 99% of patients experienced adverse effects, with 88% of patients developing CRS and 15% developing neurotoxicity [70]. Furthermore, 58% of patients experienced any grade infection, with 28% experiencing grade 3 or higher infection. Similar results are seen following treatment with BisAbs - in the MajesTEC-1 trial, out of 165 RRMM patients who received teclistamab, 76.4% of patients experienced infection of any grade, with 13% patients dying from grade 5 infection [78]. This significant risk of infection can be partly attributed to poor medullary reserve following previous therapies but is largely due to the effects of redirected T-cell therapy itself. Neutropenia, lymphopenia and hypogammaglobulinemia secondary to B-cell aplasia can be seen; as BCMA is expressed on healthy plasma cells as well as MM cells, BCMA-targeting therapies can have on-target off-tumour effects and thus deplete the B-cell reservoir, increasing infection risk [98]. 

Infection risk is also heightened due to tissue and endothelial damage and immunoparalysis following CRS, as well as the use of steroids and anti-IL6 blockade to treat CRS – a retrospective, multicentre study of BisAb-treated patients with MM revealed that corticosteroid use for the treatment of CRS or ICANS was associated with a significantly increased risk of first infection (HR 2.13) [99]. In fact, death from infectious complications is the driver of non-relapse mortality with redirected T-cell therapies; we are thus approaching a period where a subset of older myeloma patients may be more likely to die secondary to infectious complications rather than from myeloma [98]. As a result, despite the unprecedented success of newer cellular therapies for RRMM, their use is not without risk, and patient selection should therefore be stringent. Those that are frailer, with ECOG statuses of above 2, may not be suitable candidates for switching drug class to cellular therapies - instead, they may be better suited to receiving more tolerable retreatment with drugs from the same class.

Despite these concerns, we cannot overlook the fact that results from trials such as the KarMMa-2 and CARTITUDE-4, as well as the Belantamab combination trials – DREAMM-7 and DREAMM-8 – have shown that for patients experiencing high-risk disease relapse, a change in therapeutic strategy with these new molecules could provide greater benefits compared to re-exposure to previously administered drug classes [65, 66, 70, 71]. 

Furthermore, prospective, and large real-world studies are still needed to analyse the rate of complications of these treatments as well as the feasibility of delivery in patients with comorbidities. Results from these studies give us a better understanding of safety profile and efficacy, and recommendations can be made in routine clinical practice.

## Conclusion

Newer treatment modalities for patients with MM opens a wide range of possibilities for the treatment of relapsed disease. Even though outcomes are positive in controlled trials, there is still limited real-world data and restricted world-wide approval. Therefore, a decision must be made between re-exposure to previous drugs or changing to a different mode of action during relapse, considering MM as an incurable disease, and a finite number of treatment strategies to offer to patients. Whilst achieving a deep response is one of the main clinical outcomes, offering patient a good quality of life must be a key driver for choosing the best treatment strategy.
